# Oral bisphosphonate-associated osteonecrosis of maxillary bone: A review of 18 cases

**DOI:** 10.4317/jced.51694

**Published:** 2014-12-01

**Authors:** Edgardo López-D’alessandro, Fabiana Mardenlli, Marisa Paz

**Affiliations:** 1 Departments of Stomatology I and II, School of Dentistry. Universidad Nacional de Rosario. Argentina

## Abstract

Biphosphonate-associated maxillary bone osteonecrosis (BPMO) is a complication related to nitrogen-containing biphosphonate therapy. This adverse effect occasionally appears in patients who are administered biphosphonates through intravenous infusion for the treatment of cancer involving bone metastases. It can also present, in a lesser degree, in patients who take these drugs orally for the treatment of osteoporosis. Lately, there has been an increase in the number of cases of osteopenia and osteoporosis due to the increasing life expectancy of the world’s population. In our country, a risk group composed mainly of older women who have been diagnosed with osteopenia or osteoporosis, and submitted to the continuous action of oral biphosphonates, is emerging. In this paper we present 18 cases of BPMO associated to the use of oral biphosphonates, diagnosed and treated in the Department of Stomatology of the School or Dentistry at Universidad Nacional de Rosario, Argentina. A protocol was designed in which the following information was recorded: age and sex of the patients, the original disease which led to therapy with oral biphosphonates, the drugs used and the period in which those drugs were administered, the clinical features and location of the lesions, together with triggering factors.

** Key words:**Maxillary osteonecrosis, mandibular osteonecrosis, oral biphosphonates, alendronate, ibandronate.

## Introduction

BPMO often appears, with one or several breaks in continuity, in the alveolar process mucosa, with exposure of a necrotic maxillary or mandibular bone which shows no tendency to healing. This disease can present spontaneously, or after a prosthetic-related trauma, minor dental surgery such as dental extraction or dental implant, gingivectomy, etc. There is no scarring of the wound for a minimum of 6 to 8 weeks in patients not undergoing radiation therapy who are being or have been administered intravenous or oral biphosphonates for long periods. The risk of this side effect is related to the cumulated dosage, the administration route, and the period in which biphosphonates have been administered. Specific risk factors have been detected: the intravenous administration route, the potency of the particular biphosphonate, the presence of periodontal disease, the presence of diabetes, and the concomitant treatment with systemic corticosteroids. Oral biphosphonate-associated BPMO’s incidence is very low, between 0.01 and 0.04%. The risk of new cases arising varies between 1/100,000 and 1/10,000 per year of therapy. These values appear to be increasing, especially in patients with osteoarticular pathologies like arthritis, osteopenia, and osteoporosis who are being treated with oral biphosphonates (1-10).

## Material and Methods

Eighteen cases of patients with oral biphosphonate-associated BPMO (taking alendronate or ibandronate) for the prophylaxis or treatment of osteopenia or osteoporosis were included in this study after the signature of the corresponding informed consent. They were diagnosed and treated in the Department of Stomatology, School of Dentistry, at Universidad Nacional de Rosario, Argentina, between 2009 and 2013. Cases of BPMO in patients who were administered intravenous biphosphonates for the treatment of cancer or other osseous pathologies were excluded from the study. Patients with diabetes, patients receiving concomitant therapy with systemic corticosteroids, and those who decided to not give their consent to participate were also excluded. A data collection protocol was designed, in which the following information was registered: age and sex of the patients, clinical findings and location of the lesions, type of drugs used, the dosage and interval between doses, the administration routes and the period in which the drugs were administered, the original disease which led to the use of biphosphonates, and the presence of triggering factors. This study was approved by our institution’s bioethics committee.

## Results

The median age of the patients studied was 68 years (65-85). Sex distribution showed that only one of the patients with BPMO was male, while the remaining 17 cases developed in female patients. The presence of triggering factors was confirmed in 44.4% of the patients, who associated the onset of the disease with a specific situation; 5 patients had dental extractions, 2 had prosthetic-related trauma, and 1 had undergone dental implants; none of the remaining 10 patients associated any particular factor with the onset of the disease. As for the type of drug used, it was determined that 50% of the cases were receiving only alendronate at a dose of 70 mg/week, orally ( Table 1).

**Table 1:**
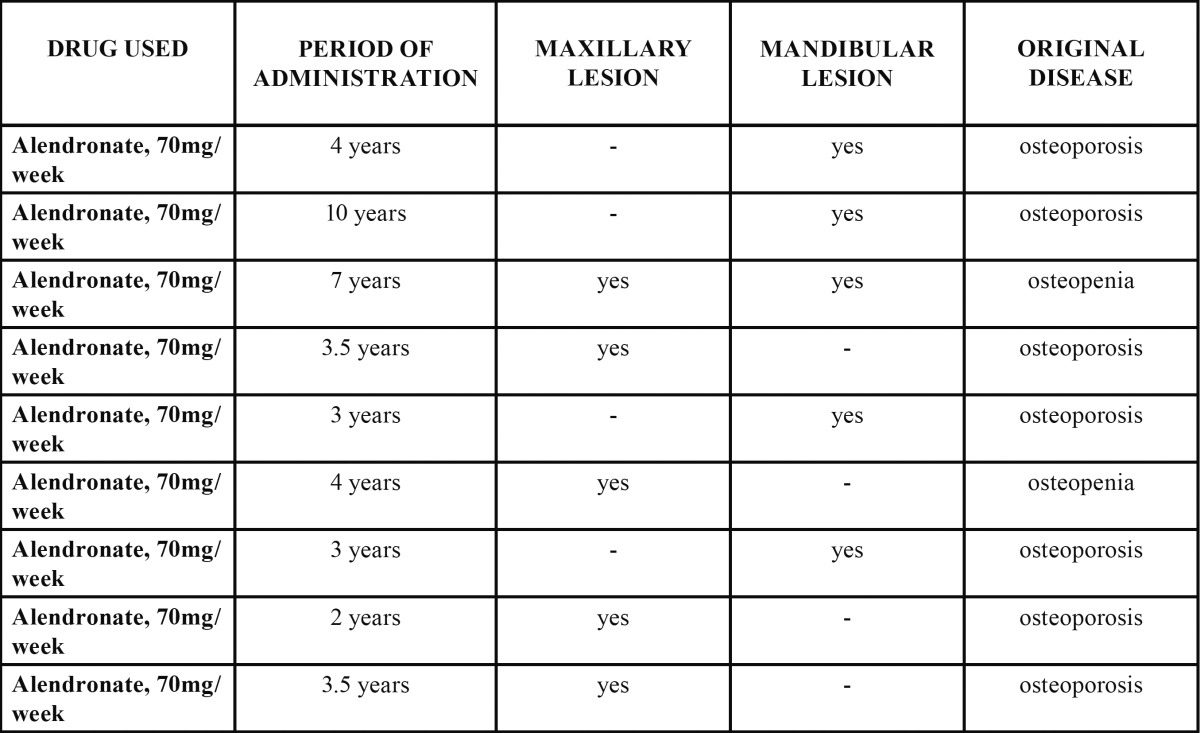
BPMO cases associated with the use of alendronate.

On the other hand, 44.4% of the patients received only 150 mg/month of oral ibandronate ( Table 2).

**Table 2:**
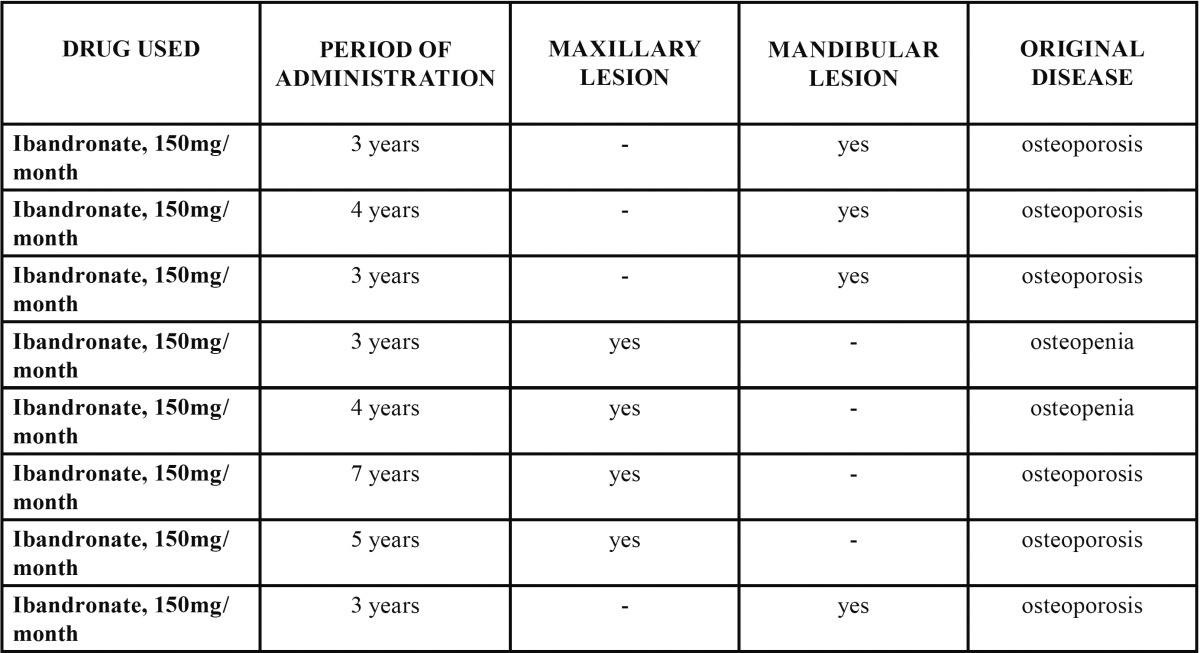
BPMO cases associated with the use of ibandronate.

It could also be established that only one patient (5.6%) received both alendronate and ibandronate concomitantly for 2 years ( Table 3).

**Table 3:**
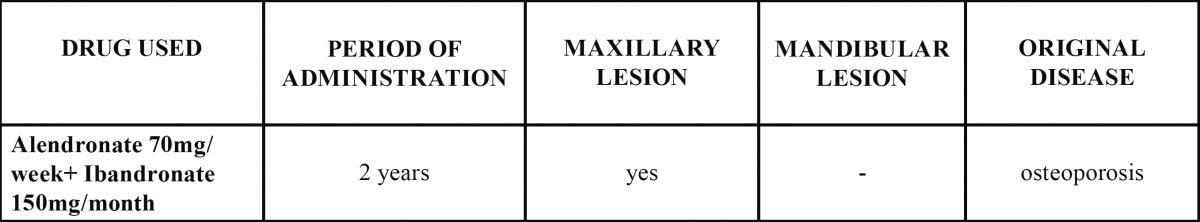
BPMO cases associated with the concomitant use of alendronate and ibandronate.

**Figure 1:**
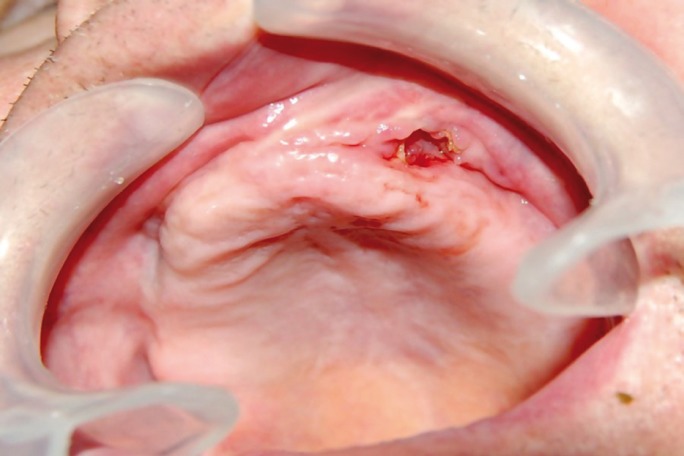
Areas of exposed necrotic bone.

**Figure 2:**
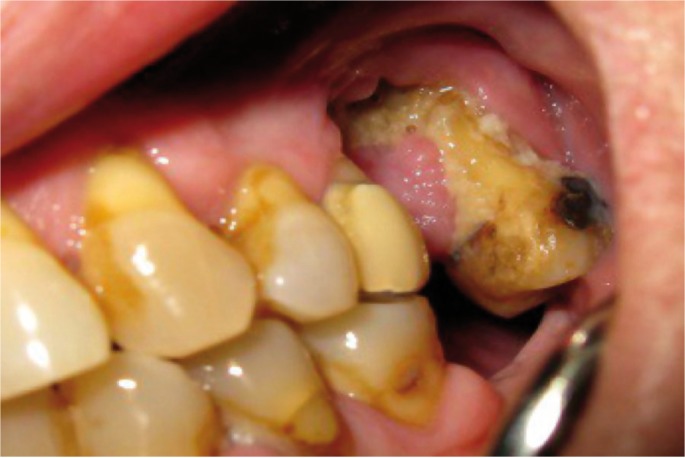
Areas of exposed necrotic bone.

**Figure 3:**
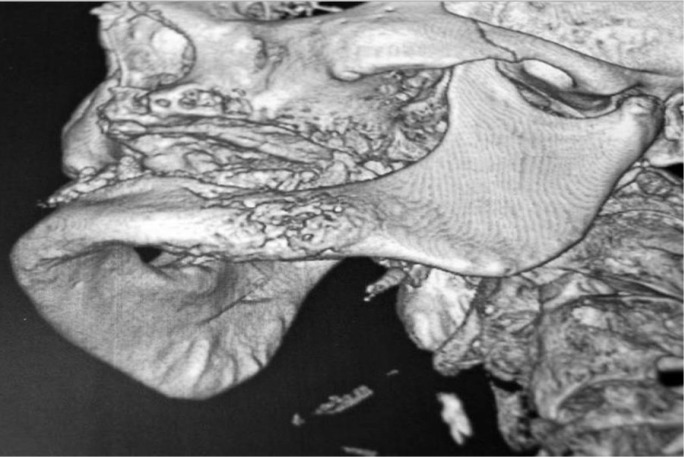
Mandibular involvement with extraoral fistula.

**Figure 4:**
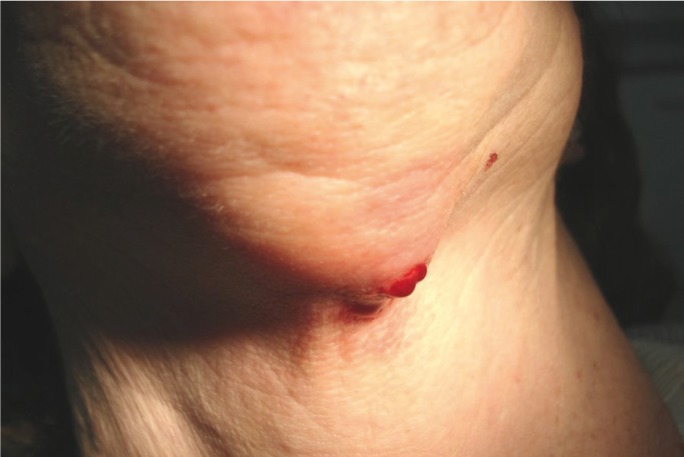
Mandibular involvement with extraoral fistula.

The period of use of biphosphonates prior to the appearance of BPMO was established, in average, as 4.11 years; this being the period of administration before the appearance of the condition. As for the original disease which required the administration of biphosphonates, it was determined that 77.8% of the patients had been diagnosed with osteoporosis, and the remaining 22.2% had osteopenia. As regards the clinical features of the lesions, most of them were areas of exposed necrotic bone of less than 2 cm, with little bleeding and slightly painful; some were also secondarily infected. (Figs. 1,2) In 94.4% of the cases only single lesions were observed, while in one of the cases (5.6%), lesions were detected both in the maxillary bone and in the mandible. In only one of the cases, an area of significant mandibular involvement, with an extraoral fistula, was found; the subject was a female patient who had received alendronate continuously for a period of 10 years (Figs. 3,4).

## Discussion

BPMO has frequently been associated with the use of intravenous biphosphonates in patients undergoing treatment for hypercalcemia of malignancy or cancer with bone metastases; its incidence being very low for oral biphosphonates. Over the last few years, the increase in the use of oral biphosphonates for the treatment and prophylaxis of osteoporosis has led to an increase in the number of described cases of BPMO associated to the oral use of these drugs. This is a very disturbing fact, since the population suffering from osteoporosis increases every year, and it can be expected the amount of patients developing BPMO within this group to rapidly rise. Junquera *et al.* (3) noticed an increase in BPMO cases in patients with certain pathologies like rheumatoid arthritis, in which the development of serious osteoporosis had required treatment with oral biphosphonates. This was also observed in all of our patients. In a systematic review of 368 cases of BPMO, Woo *et al.* (1) observed that the oral biphosphonate which more frequently caused BPMO was alendronate, as we also noted in our own review. Lazarovici *et al.* (2) studied 27 patients presenting with BPMO, concluding that the median time of emergence of the disease was 60 months for patients who had taken alendronate, 13 months for those who had taken zolendronic acid, and 35 months for those who had taken pamidronate. A review of 20 cases of BPMO conducted by Bagán *et al.* (11) showed an average of 27.3 months for intravenous biphosphonates. In a study of 44 cases, Bocanegra *et al.* (12) determined that the average time of appearance of BPMO in patients who took alendronate was 7 years. In our study, the mean time of onset was 49.32 months for the total of patients studied, who took alendronate, ibandronate, or a combination of both. Bagán *et al.* (13) informed of a group of 10 patients who presented with posterior mandibular osteonecrosis after receiving chemotherapy for the treatment of cancer. In 50% of the cases, the osteonecrosis was located in the mandible, and in most of the cases the lesion appeared after previous tooth extractions. Marín *et al.* (14) published a series of 5 cases of BPMO associated to the intake of oral biphosphonates, with a collective history of dental manipulation at the time of the diagnosis of BPMO. In our series, over 18 cases reviewed, 10 patients presented with lesions in the mandible; in 5 of these patients the lesions were preceded by dental extractions, in 2 cases by prosthetic-related trauma, and in 1 case by dental implants. Ruggiero *et al.* (10) mention 7 cases of BPMO related to osteoporosis from a total of 63 patients studied who had BPMO, none of which had a history of malignant lesions or previous chemotherapy. We present 18 cases of BPMO in patients undergoing therapy with oral alendronate or ibandronate, for the treatment of osteopenia or osteoporosis, with no history of malignancy or chemotherapy. Bocanegra *et al.* (15) reported 3 cases of BPMO in diabetic female patients who took oral alendronate, and were submitted to dental extractions without proper antibiotic prophylaxis. Diabetic patients were excluded from our study. In 2012, Diniz *et al.* (16) informed of 20 cases of BPMO in 19 female and 1 male patients who took oral biphosphonates (alendronate or ibandronate) with certain similarities with our cases. Today, a great number of patients take oral biphosphonates worldwide. In Argentina, we observe a marked increment of BPMO in patients who take oral biphosphonates for the prophylaxis or treatment of osteoporosis. When these people need dental care, we should consider the possibility of them developing BPMO, especially when they require dental treatment which includes minor surgery (dental extractions, gingivectomy, biopsy or dental implants). Likely prescribers of oral biphosphonates (hematologists, traumatologists, rheumatologists, gynecologists, oncologists, etc.) should assess the possibility of appearance of this adverse effect at the time of choosing what to prescribe. In case there was no alternative, the patient should be informed of the risk of developing BPMO, and of the importance of taking care of their oral health with regard to their treatment. The present study does not state any conflict of interests.
